# Cancer Therapy Due to Apoptosis: Galectin-9

**DOI:** 10.3390/ijms18010074

**Published:** 2017-01-01

**Authors:** Koji Fujita, Hisakazu Iwama, Kyoko Oura, Tomoko Tadokoro, Eri Samukawa, Teppei Sakamoto, Takako Nomura, Joji Tani, Hirohito Yoneyama, Asahiro Morishita, Takashi Himoto, Mitsuomi Hirashima, Tsutomu Masaki

**Affiliations:** 1 Department of Gastroenterology and Neurology, Faculty of Medicine, Kagawa University, Ikenobe 1750-1, Miki, Kita, Kagawa 761-0793, Japan; 92m7v9@med.kagawa-u.ac.jp (K.F.); oura@med.kagawa-u.ac.jp (K.O.); tadokoro@med.kagawa-u.ac.jp (T.T.); samukawa@med.kagawa-u.ac.jp (E.S.); sakamoto@med.kagawa-u.ac.jp (T.S.); nomura@med.kagawa-u.ac.jp (T.N.); tani@med.kagawa-u.ac.jp (J.T.); yoneyama@med.kagawa-u.ac.jp (H.Y.); morishita@med.kagawa-u.ac.jp (A.M.); 2 Life Science Research Center, Kagawa University, Ikenobe 1750-1, Miki, Kita, Kagawa 761-0793, Japan; iwama@med.kagawa-u.ac.jp; 3 Department of Medical Technology, Kagawa Prefectural University of Health Sciences, Hara 281-1, Mure, Takamatsu, Kagawa 761-0123, Japan; himoto@chs.pref.kagawa.jp; 4 Department of Immunology and Immunopathology, Faculty of Medicine, Kagawa University, Ikenobe 1750-1, Miki, Kita, Kagawa 761-0793, Japan; mitsuomi@kms.ac.jp

**Keywords:** galectin-9, glycan, apoptosis, pyroptosis, caspase-1, cancer, therapy

## Abstract

Dysregulation of apoptosis is a major hallmark in cancer biology that might equip tumors with a higher malignant potential and chemoresistance. The anti-cancer activities of lectin, defined as a carbohydrate-binding protein that is not an enzyme or antibody, have been investigated for over a century. Recently, galectin-9, which has two distinct carbohydrate recognition domains connected by a linker peptide, was noted to induce apoptosis in thymocytes and immune cells. The apoptosis of these cells contributes to the development and regulation of acquired immunity. Furthermore, human recombinant galectin-9, hG9NC (null), which lacks an entire region of the linker peptide, was designed to resist proteolysis. The hG9NC (null) has demonstrated anti-cancer activities, including inducing apoptosis in hematological, dermatological and gastrointestinal malignancies. In this review, the molecular characteristics, history and apoptosis-inducing potential of galectin-9 are described.

## 1. Introduction

Physiologic tissue development and normal kinetics of mature tissues depend on apoptosis to remove excess or abnormal cells from normal tissue. These processes allow the tissue to maintain its normal morphological quantity and physiological quality [1]. Abrogation of apoptosis results in an imbalance of mitosis and cell death in the tissue, disturbing tissue homeostasis, while accumulating functionally impaired cells that cannot undergo apoptosis. The accumulation of excess cells causes hyperplasia and the increase of functionally dysregulated cells, which might also be genetically impaired, contributes to carcinogenesis [2]. A series of studies prior to the proposal of “apoptosis” in the 1970s analyzed the contribution of mitosis and cell death to the tumor doubling time, reporting that cell death appears to determine tumor growth more so than mitosis [3,4,5].

Lectin, defined as “a carbohydrate-binding protein that is not an enzyme or antibody”, has been the subject of research on anti-cancer agents for nearly a century. In fact, lectin can affect cell fate, as expected from the variety of lectin types in animal, their wide distribution in various tissues and differential expression from embryonic cells to malignant cells [6]. Soluble endogenous lectins, represented by galectins among animal lectins, interact extracellularly with cell surface glycoconjugates and transmit signals that potentially determine cell fate. On the other hand, intracellular lectins that are located in plasma membranes modulate such signals by regulating receptor-ligand interactions, receptor trafficking and endocytosis [7,8,9,10].

Among diverse animal lectins, galectin-9, the ninth member of the β-galactoside-binding soluble lectin family, was independently cloned by four groups in 1997 [11,12,13,14]. Galectin-9, which is expressed on thymocyte surfaces, has been revealed to induce T lymphocyte apoptosis in thymic negative selection of T cells, playing a pivotal role in the development of the acquired immune system [12]. Furthermore, subsequent studies clarified that the administration of exogenous galectin-9 induced apoptosis of human malignant cells and immune responsible cells in vitro and in vivo*,* suggesting that galectin-9 can be a candidate anti-cancer agent based on the carbohydrate recognition function of this animal lectin [15,16]. In this review, malignant cell apoptosis and galectin-9 are reviewed and the potential for galectin-9 to be an anti-cancer agent is presented.

## 2. Apoptosis and Malignancy

### 2.1. Apoptosis, a Type of Programmed Cell Death

The idea of programed cell death was first proposed in 1972 based on morphological profiling and differentiation from necrosis [1]. Apoptosis, a type of programmed cell death, is characterized by cytoplasmic cell shrinkage, chromatin condensation and DNA fragmentation, and segmentation into apoptotic bodies (called blebbing) and occurs in response to extrinsic and intrinsic stimuli [17]. The plasma membrane is kept intact in apoptosis, preventing inflammation and tissue injury that would otherwise occur in response to cytokine release from dying cells.

Apoptosis, which was first defined by morphological investigations, has been further investigated in terms of the associated biochemical aspects [18]. Two protein families, the caspase and Bcl-2 families, play crucial roles in executing or halting apoptosis. Caspases are cysteine-dependent, aspartate-specific peptidases, which initiate or affect apoptotic and inflammatory pathways. In humans, a total of 11 caspases, the 1st to 10th and 14th, have been cloned [19]. The Bcl-2 protein family shares homology domains between members. Bcl-2 and its relatives have four domains, BH1-4. They are divided into two subfamilies; anti-apoptotic proteins represented by Bcl-2 are Bcl-2, Bcl-x_L_, Bcl-w, Mcl-1, A1 and Bcl-B and pro-apoptotic proteins are Bax and Bak. Other Bcl-2-relatives, BH3-only proteins, have a single homology domain (BH3) [20]. BH3-only proteins include at least eight members: Bik, Bad, Bim, Bmf, Hrk, Noxa and Puma. BH3-only proteins have pro-apoptotic effects by interfering with anti-apoptotic Bcl-2 relatives or directly stimulating pro-apoptotic Bax and Bak [21,22].

**Figure 1:**
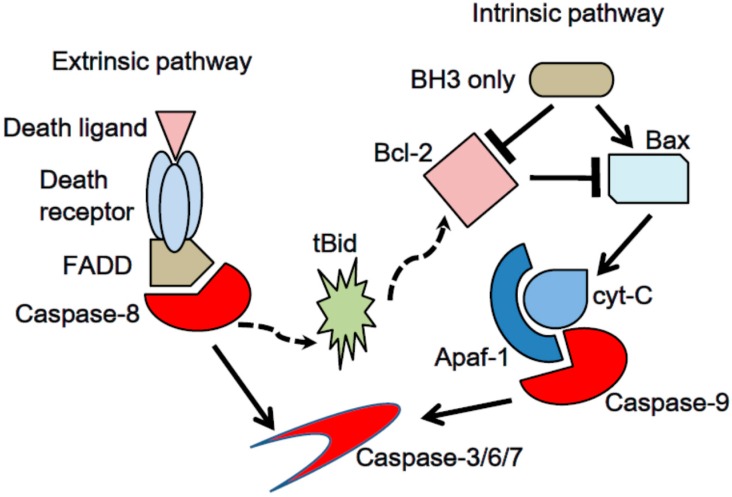
Two major pathways of apoptosis. Extrinsic pathway is induced by death ligand-death receptor interaction. Oligomerized death receptors form death inducing signaling complex (DISC). DISC activates initiator pro-caspase-8/10. Intrinsic pathway is initiated by Bcl-2 family members. Pro-apoptotic proteins, Bax and Bak, lead mitochondrial outer membrane permeability (MOMP), resulting leakage of cytochrome-c from mitochondrial inter membrane space into cytosol. Thus, cytochrome-c constructs apoptosome with Apaf-1 and activates initiator pro-caspase-9. Caspase-8 in extrinsic apoptotic cascades cleaves Bid into tBid, which recruits intrinsic apoptotic response.

Apoptosis is classified into two subtypes according to the biochemical machineries: extrinsic and intrinsic, as shown in Figure 1. The extrinsic apoptotic mechanism is activated by the interaction between death receptors on the cell surface and their ligands [23]. The combination of death receptor-ligands is represented by Tumor Necrosis Factor (TNF) Recptor-1-TNFα, FAS-Fas Ligand (FasL), TNF-related apoptosis-inducing ligand (TRAIL) Receptor-1 and -2-TRAIL. Death receptors share a structural feature in their intracellular components, called the death domain. Upon stimulation, death domains enable death receptors to form an oligomer and change conformation. Then, death receptors and adapter proteins, such as Fas-Associated protein with Death domain (FADD) and TNF Receptor-1 associated Death Domain protein (TRADD), construct a complex called death-inducing signaling complex (DISC), which activates initiator pro-caspase-8/10 [24].

The intrinsic apoptotic pathway is characterized by mitochondrial change in response to various stress signals, such as severe genetic damage, hypoxia and oxidative stress, which activate the initiator pro-caspase-9 [25]. Mitochondrial pro-apoptotic proteins, BH3-only members, antagonize anti-apoptotic proteins, Bcl-2, Bcl-x_L_ and Mcl-1. Subsequently, the mitochondrial outer membrane is disrupted, and its permeability increases, resulting in cytochrome-c leakage into the cytosol [26]. Cytochrome-c in cytosol forms a complex with Apaf-1, called the apoptosome, which assists in auto-activation of initiator pro-caspase-9.

Extrinsic and intrinsic apoptosis share the executor caspase-3/6/7, which cleave critical cellular components and induce cell death [27]. Extrinsic apoptotic cascades recruit the intrinsic pathway via one BH3-only protein, Bid, when the cell does not die from death receptor-ligand interaction alone [2]. Caspase-8 is subsequently activated through death receptor-ligand interactions and cleaves Bid, generating a tBid fragment, resulting in mitochondrial outer membrane permeability (MOMP) in the intrinsic pathway.

### 2.2. Evasion of Apoptosis: A Hallmark of Cancer

In general, malignant tissues are characterized by six cell biology features as follows: (1) self-sufficiency in growth signals; (2) insensitivity to growth-inhibitory signals; (3) evasion of cell death; (4) limitless replication potential; (5) sustained angiogenesis; and 6. tissue invasion and metastasis [28]. In combination with other cancer hallmarks described above, evasion of apoptosis affects tumor growth kinetics through decreasing the elimination of DNA-damaged cells, resulting in aberrant cellular proliferation [1]. Furthermore, dysregulation of apoptotic machineries provides the basis for tumor cell chemoresistance [21,29]. Therefore, apoptosis promotion is an essential anti-cancer strategy in addition to the suppression of cancer cell mitosis.

### 2.3. Glycans Differentiate Apoptotic Signals and Affect Cell Fate

Cell surface receptors which commit to apoptotic process are glycosylated. Protein glycosylation alters protein structure, conformation and function [30]. The glycan structure is diversified by 10 monosaccharide species that combine to generate a glycan according to differences in the linkages, aromatic status, branching, lengths and substituted components [31]. Glycan-protein binding also creates a variety of glycoconjugates. A glycan can covalently bind to at least nine amino acid types. Two representative linkages are *N*-glycosylation binding to aspargine residues and *O*-glycosylation to serine/threonine residues [32,33]. Proteomic information is thus modulated and diversified by exponentially differentiated structure of glycoconjugates, that is, glycome. Different from DNA synthesis, RNA transcription or Protein translation, glycan lacks DNA template. Otherwise, glycosylation is influenced by non template factors such as carbohydrate substrates availability, activity and expression level of enzymes and transporters [34].

Aberrant glycosylation of death receptors alters apoptotic pathways, modifying cell fate. For example, two *N*-glycosylation sites on the Fas ligand stabilize the extrinsic apoptotic machinery DISC [35]. The stability of DISC, DISC-DISC interactions and pro-caspase-8 oligomerization are important to the initiation of extrinsic apoptosis. If Fas ligands are aberrantly glycosylated, Fas does not oligomerize or induce apoptosis [36]. For TRAIL Receptor-1 and -2, which have two potential *O*-glycosylation sites, fucosylation of core-2 *O*-glycans increases the sensitivity of cancer cells to TRAIL-induced apoptosis [37,38]. Thus, glycosylation of molecules that are pivotal to initiating apoptosis might modulate apoptotic signal transduction by strengthening or weakening the affinity of receptors for their ligands.

### 2.4. Lectins Regulate Diverse Information on Glycoconjugates

With consideration for the role of glycosylation on apoptotic machinery, lectin as a glycan ligand can regulate apoptotic pathways and modulate cell fates [39]. Lectin, glycan-binding proteins, multivalently interact with glycosylated death receptors, which might alter the affinity of pro-apoptotic molecules for their ligands [40,41,42]. Lectin in the plasma membrane forms lattice structures by binding to and bridging between glycoconjugates. The lattice structure might help anchor receptors on the cell surface, assigning a proper location for the receptors on the cell surface and halting receptor turnover [43].

The galectin family is a group of β-galactoside-specific soluble animal lectins that share highly conserved sequences of carbohydrate recognition sites. Recent studies have revealed that galectin members can promote or suppress apoptotic pathways, which contributes to acquired immunity through the negative selection of T lymphocytes in the thymus [12]. Endogenous galectins also orchestrate and play pivotal roles in tumor progression, in which some galectins are upregulated, and others are downregulated [44]. Furthermore, one of recombinant-type human galectin-9, hG9NC (null), which has greater protease-resistant properties than endogenous original molecules, has demonstrated anti-cancer activity, inducing apoptosis on epithelial cancer species and hematological malignancies.

## 3. Identification of Galectin-9

**Figure 2:**
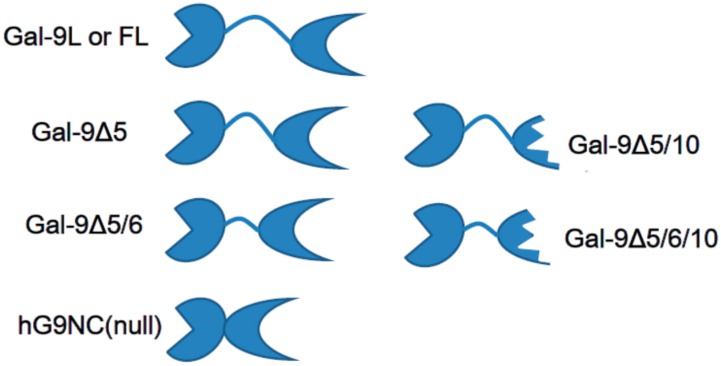
Structure of galectin-9 isoforms and recombinant galectin-9 lacking a linker peptide, human recombinant galectin-9 (hG9NC) (null). Galectin-9 has two distinct carbohydrate recognition domains connected by a linker peptide with variable lengths. Two isoforms lacking exon 10 transcripts are not equipped with the C-terminal carbohydrate recognition domain.

Galectin-9, a 36-kDa β-d-galactoside mammalian lectin, was identified from murine embryonic kidney and human Hodgkin’s lymphoma tissues in 1997 [11,12]. The galectin exhibited specific affinity to the lactosyl group and had two distinct carbohydrate recognition domains in the N-terminus and C-terminus. The two domains were connected by a linker peptide, such as galectin-4, -6 and -8, which are tandem repeat-type peptides. The linker peptide length varied among the galectins and yielded three isoforms (Figure 2). This galectin was called galectin-9. The existence and characteristics of galectin-9 have been investigated since 1991. ConA-activated CD4^−^ T lymphocytes produced eosinophil chemoattractants, called ecalectin, which has a molecular weight of 30–40 kDa; no hydrophobic signaling peptides had to be secreted outside the cells [45,46]. Although ecalectin was first considered a variant of galectin-9 because the difference in the sequence was limited to only 4 amino acids, it was later found to be identical to the medium-sized isoform of galectin-9, and the difference in the sequence was attributed to sequencing errors [14,47]. The urate transporter was also cloned and found to be identical to galectin-9 in humans and in mice [13,48]. Amino acid sequencing of the urate transporter revealed no differences from long isoform of galectin-9. Four amino acid differences that were caused by 4 nucleotide differences between the human urate transporter and galectin-9 (Genbank accession number Z49107) were attributed to sequencing errors in galectin-9.

## 4. Molecular Profiling of Galectin-9

### 4.1. Genomic Localization and Expression

The galectin-9 gene *LGALS9* is encoded on the short arm of chromosome 17, 17q11.2 (HGNC:6570), which consists of 11 exons, yielding a 355-amino acid-length product [9]. So-called galectin-9 protein, which has several isoforms, is presumed to be transcribed from the genetic location, *LGALS9*. On the 17q11.2, two transcripts that are almost identical to the nucleotide sequence galectin-9, *LGALS9B* (HGNC:24842) and *LGALS9C* (HGNC:33874), are encoded, indicating gene duplication. Whether the two galectin-9-like molecules are expressed and active remains controversial.

### 4.2. Structure

Galectin-9 is classified as a tandem-repeat type galectin, which consists of two distinct β-galactoside-binding sites linked by a peptide [12]. The N-terminal carbohydrate recognition domain (CRD) of 148 amino acids shares 39% of its sequence with the C-terminal CRD of 149 amino acids [11]. X-ray crystallography indicated that N-CRD is formed by six-stranded (S1–S6) and five-stranded (F1-F5) β-sheets as well as a short α-helix with β-sandwich motif [49] The C-CRD structure, as for N-CRD, consists of two anti-parallel S1-S6 β-sheets and F1-F5 β-strands with an α-helix [50]. Both N- and C-CRD have carbohydrate-binding pockets with S4, S5 and S6 β-strands, which differ in amino acid sequence from each other, resulting in differential affinity to β-galactosides and distinct physiologic activities [51,52]. However, eosinophil chemoattraction by galectin-9 depends on both of two CRDs in the single molecule, although N-CRD and C-CRD can attract eosinophils [47].

Linker peptides flexibly connect two distinct carbohydrate recognition domains. The CRDs are able to rotate and thus have potential to constitute more diverse glycan-galectin-9 complexes. Galectin-9 has protease-sensitive sites in linker peptides and is cleaved into two monovalent sugar-specific proteins that are similar to the galectin prototype [53].

Galectin-9 has three classical isoforms, according to different lengths of a flexible linker peptide, as a result of posttranscriptional splicing (Figure 2). The linker peptide of 58 amino acids represents the long-sized isoform (gal-9L or gal-9FL). The linker peptides with 26 and 14 amino acids represent medium-sized (gal-9M) and short-sized (gal-9S) galectin-9 [54]. The medium-sized galectin-9 lacks exon 5 (gal-9Δ5) and short–sized galectin-9 lacks exons 5 and 6 (gal-9Δ5/6). These linker peptides were not essential in some CRD-dependent physiologic actions, such as in the chemoattraction of eosinophils and the induction of T-lymphocyte apoptosis because even a recombinant galectin-9 that lacks a linker peptide had similar activities [53,54,55]. On the other hand, the linker peptide length can affect the structure of galectin-9-sugar oligomerization and lattice formation on the cell surface [56].

Furthermore, other splice variants that involve deletion of exon 10 transcripts have been reported in human samples [48,57,58]. Deletion of exon 10 transcripts from mature galectin-9 messenger RNA results in truncation of C-CRD, which is encoded on exon 11 [9]. C-CRD truncation directly affects the carbohydrate specificity of galectin-9 in each isoform. Functional diversity of galectin-9 isoforms according to the linker peptide length and C-CRD truncation has yet to be elucidated.

### 4.3. Secretion and Solubility

The secretion mechanisms of galectin members have not been well established, in part because galectins, including galectin-9, lack hydrophobic signal peptides that can enter the endoplasmic reticulum (ER) and be secreted outside cells via the classical secretion pathway [59]. While up to 50% of galectin-9 is recovered with the soluble elements of cytoplasm and another fraction with the cell surface membrane and nucleus, some evidence has clarified that galectin-9 is released from cells via the non-classical pathway, including the exosome. For example, the Jurkat T cell line that expressed galectin-9 on its cell surface released eosinophil chemoattractants, including medium- and long-sized galectin-9 isoforms, when antigens stimulated the T cells [60]. Murine CD4^+^ T cells expressing galectin-9 on their cell surface secreted soluble galectin-9, which increased upon TCR stimuli [61]. Exosomes containing galectin-9 were released from EBV-infected nasopharyngeal carcinoma cells into culture media [62,63].

### 4.4. Carbohydrate Specificity

Galectin-9 presents striking affinity for branched *N*-glycans and repeated oligolactosamines [64]. The affinity of this galectin for *N*-glycans depends on the glycan valency, with triantennary *N*-glycans interacting with galectin-9 more tightly than the biantennary *N*-glycans, and the biantennary *N*-glycans binding more tightly than the monovalent *N*-glycans. The affinity for oligolactosamines is also drastically enhanced when the repeat number is larger. In addition, the affinity for *N*-glycans and oligolactosamines is shared by N-CRD and C-CRD. Differential specificity of the two distinct CRD to glycoconjugates is also determined. N-CRD, not C-CRD, binds to Forssman pentasaccharide, A-hexasaccharide and several glycolipid-type glycans. Comprehensive data of galectin-9’s ligands are available in data repositories at Consortium for Functional Glycomics (http://www.functionalglycomics.org/).

### 4.5. Bivalency, Cross-Linking and Lattice Formation

**Figure 3:**
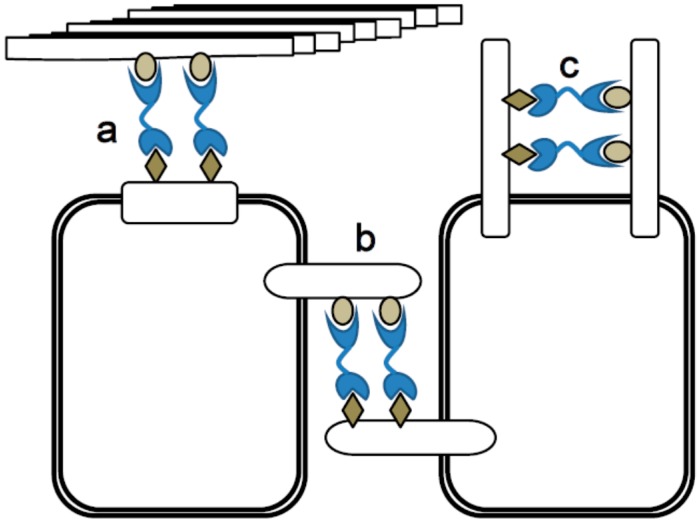
Galectin-9 functions by its divalent sugar binding activity. (**a**) Cell to extracellular matrix adhesion; (**b**) Cell to cell adhesion; and (**c**) Clustering cell surface molecules and modulate their signal delivery.

**Figure 4:**
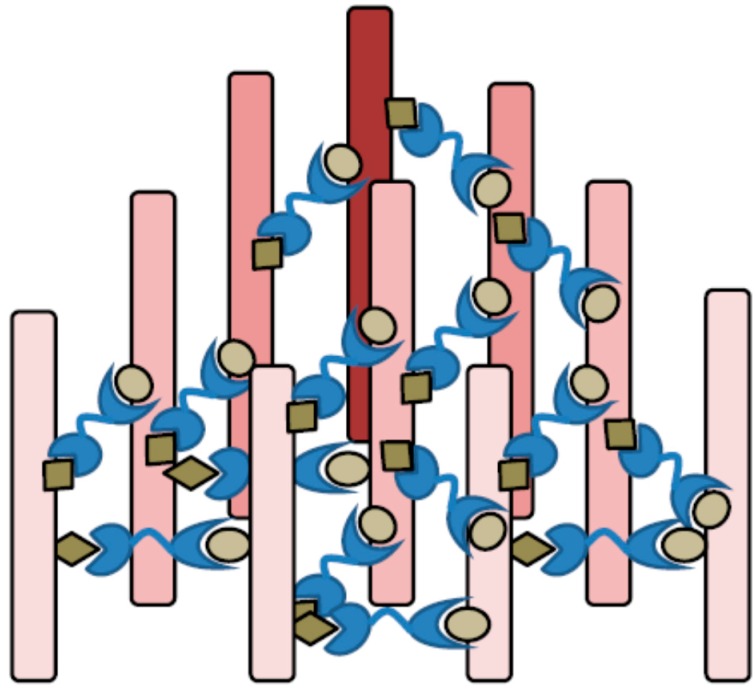
Lattice formation by galectin-9 and multivalent glycoconjugates. The galectin-9-glycan lattice provides higher diversity based on the combination of 10 monosaccharide species on glycoconjugates, different sugar-affinity of galectin-9’s two distinct carbohydrate recognition domains (CRDs) and the rotational freedom of the CRDs that is allowed by flexible linker peptides. The galectin-glycan lattices could perform three major roles in cell biology, including organizing cell membrane domains, determining thresholds of cell signaling and restricting the receptor residency time on the cell surface.

Galectin-9, a tandem-repeat type of galectin, shows bivalency and can cross-link two glycoconjugates. Thus, galectin-9 is able to execute cell to cell adhesion and cell to matrix interaction, contributing to physiologic tissue functions and pathologic responses. On cell surface, galectin-9 might cluster cell surface receptors and modulate strength and duration of signaling (Figure 3). When the glycoconjugates have bi- or multivalent potential to bind to lectins, galectin-9 and its binding partners can form a two- or three-dimensional lattice structure, as shown in Figure 4 [65]. The galectin-9-glycan lattice provides higher diversity based on the differences in the binding partners for two distinct CRDs and the rotational freedom of the CRDs that is allowed by flexible linker peptides. In general, lectin-glycan lattices are constructed by multiple low-affinity interactions and are thus diverse based on differential glycosylation of proteins or lectin expression levels [66]. The galectin-glycan lattices could perform three major roles in cell biology, including organizing cell membrane domains, determining thresholds of cell signaling and restricting the receptor residency time on the cell surface [67]. The function of lattice formation specific to galectin-9 has yet to be investigated, while that of galectin-1-glycan lattices has been well established [56,68,69].

### 4.6. Expression and Distribution

Galectin-9 distributes among tissues, such as in the liver, small intestine, thymus, kidney, spleen, lung, cardiac and skeletal muscle [70], brain [13], placenta, pancreas, prostate and colon [48]. To be specific to the cell type, this lectin is dominantly detected in leukocytes that are responsible for innate and acquired immunity [71], thymocytes [70], activated endothelial cells [72,73] and fibroblasts stimulated by IFN [74].

To be specific to malignant tissues, galectin-9 expression on the hepatocellular carcinoma (HCC) cell surface is reportedly decreased [75] as with other solid tumors, such as prostate cancer [76], cervical cancer [77] and skin cancer [78,79]. Galectin-9 expression is enhanced in comparison to that of normal adjacent tissues in oral cancer [80], pancreatic cancer [81], and hematologic malignancies [11]. For gastric cancer, the results seem controversial because the messenger RNA of galectin-9 decreased [82], while the protein expression increased compared with normal mucosa [83].

## 5. Exogenous Galectin-9 and Apoptosis

### 5.1. Apoptosis in Thymocytes and Immune Cells

Several full-length clones of recombinant galectin-9 were established from humans and mice and applied on thymocytes and immune cells, such as eosinophils, T lymphocytes, B lymphocytes, and macrophages, which underwent apoptosis [59,70,84,85]. T helper type 1 (T_H_1) cells express a counter receptor for galectin-9, Tim-3, when T cells fully differentiate from naïve cells to T_H_1 cells [86]. Galectin-9-Tim-3 interaction leads to mature T_H_1 cell apoptosis with intracellular calcium flux and cell aggregation, downregulating T_H_1 immunity [85]. Lactose antagonizes binding between galectin-9 and Tim-3, indicating that the galectin-9 stimuli on Tim-3 depend on the interaction between the galectin-9 carbohydrate recognition domain and Tim-3 β-galactoside domain.

CD8^+^ T cells and the CD4^+^ population are susceptible to exogenous galectin-9 stimuli and undergo apoptosis based on the calcium-calpain-caspase-1 pathway [59]. Galectin-9 also induces apoptosis of B cells, which do not express Tim-3 on cell surface. Although apoptosis is executed by the caspase family and three subfamilies of Bcl-2, caspase-1 is plays a more prominent role in pyroptosis than in apoptosis. Pyroptosis definitely differs from apoptosis because pyroptosis evokes inflammation, while apoptosis does not [87,88]. “Pyroptosis” was established in 2001 [89]. This programmed cell death observed in macrophages and dendritic cells is found in infection and inflammatory situations [90,91]. Recent studies have indicated that caspase-1 plays a pivotal role in apoptosis in inflammatory situations [92,93].

### 5.2. Human Recombinant Galectin-9 without a Linker Peptide, hG9NC(null)

Human recombinant galectin-9 that lacks an entire linker peptide region between the two distinct carbohydrate binding sites, which are the most susceptible in this molecule to proteolysis in sera, was designed and named hG9NC(null), as shown in Figure 2 [53]. A lack of linker peptides decreases the rotational freedom of CRDs, which might limit the lattice formation diversity by this tandem-repeat type of lectin [56]. hG9NC(null) is free from proteolytic cleavage into two monovalent lectins, which might work as antagonists against the CRD-dependent effects of original divalent galectin-9.

### 5.3. Malignant Cell Apoptosis Induced by the Recombinant Galectin-9 hG9NC(null)

Recombinant galectin-9 induces apoptosis of malignant cells, such as hematologic malignant cells [15,16], malignant melanoma [94] and gastrointestinal tumor species [95,96,97,98], in vitro and in vivo. Galectin-9 directly activates apoptotic cell deaths to these cell lines in vitro, not via activation of tumor immunity, demonstrating that the intracellular mechanisms of apoptosis vary depending on cell lines and that apoptosis common pathways have not previously been established [99]. In an in vivo study, galectin-9 modulates tumor immunity and suppresses tumor progression as well as directly binds to cell surface glycans and presents direct apoptotic effects.

**Figure 5:**
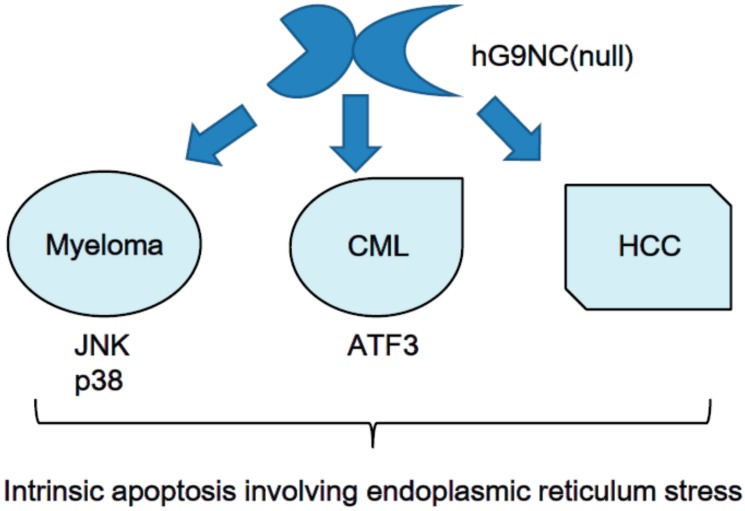
Human recombinant galectin-9 induces intrinsic apoptosis of cancer involving endoplasmic reticulum. The receptors for galectin-9 lacking a linker peptide, hG9NC (null), in the three malignancies remain unknown.

For hematologic malignancies, galectin-9 induces the apoptosis of chronic myelogenous leukemia (CML) cells [15] and myeloma cells [16]. For CML cells, galectin-9-dependent apoptosis involves intrinsic apoptotic pathways, inducing ATF3 expression, and does not depend on death receptors. Myeloma cells undergo galectin-9-induced intrinsic apoptosis through JNK and p38 MAP kinase pathways. Apoptosis of CML and myeloma is complicated with endoplasmic reticulum stress. Counter-receptors for galectin-9 on the CML or myeloma cell surface have not been clarified yet. The interaction between galectin-9 and its receptor on myeloma cells likely depends on the carbohydrate-recognition function of this lectin because lactose antagonizes its apoptotic effect (Figure 5).

Several cell lines of malignant melanoma, which does not form colonies when it proliferates, are aggregated and driven to apoptosis [94]. Intracellular pathways, including cell surface receptors for malignant melanoma cell apoptosis, have remained undefined.

Among gastrointestinal cancers, HCC lines have apoptotic responses to galectin-9 stimuli [95]. HCC apoptosis also belongs to intrinsic pathways, including endoplasmic reticulum stress, and it is not affected by death receptors. Tim-3 was not detected on the HCC cell surface, but the genuine receptor should be glycosylated by β-galactoside because lactose suppressed galectin-9-induced apoptosis. Cholangiocellular carcinoma, gallbladder cancer and gastric cancer are subject to galectin-9-induced apoptosis [96,97,98].

The pharmacokinetics of galectin-9, when subcutaneously and intraperitoneally injected into a mouse, are well established [100]. When 10 μg of galectin-9 was injected into a murine body, the Cmax was 35 ng/mL for subcutaneous injection and 61 ng/mL for intraperitoneal injection, whereas the minimal dose of galectin-9 for demonstrating pharmacological effects was as little as 1 μg/mouse, and no significant side effects were reported. While the physiological median value of galectin-9 in human sera is low-to-undetectable [101], the pharmacokinetics of exogenous galectin-9 in humans remain to be investigated. Additionally, the dose at which this artificial galectin evokes pharmacological effects with or without side effects in humans has yet to be elucidated. In the series of reports described above, the serum concentration of recombinant galectin-9 was determined by ELISA using polyclonal antibodies generated by injection of a recombinant peptide that corresponds to the COOH-terminal domain of the human protein into rabbits [101,102,103,104]. The galectin-9 concentration, measured by ELISA, might be affected by antibody clones.

## 6. Conclusions

Galectin-9 directly induces malignant cell apoptosis, although malignancy partially depends on dysregulation of apoptotic mechanisms. A protease-resistant galectin-9, which lacks a linker peptide, performs apoptotic activities because the interaction between galectin-9 and its receptor depends on the carbohydrate recognition domain of lectin. However, the pharmacological kinetics of galectin-9 in humans have not yet been assessed. According to in vitro work, the cell surface receptor for galectin-9 in cancer apoptosis has not been identified, and intracellular pathways processed in apoptosis remain to be thoroughly investigated. Galectin-9 has potential as an anti-cancer agent, but these questions should be resolved before drawing major clinical conclusions.
